# CD4^+^ T Cells Play a Critical Role in the Generation of Primary and Memory Antitumor Immune Responses Elicited by SA-4-1BBL and TAA-Based Vaccines in Mouse Tumor Models

**DOI:** 10.1371/journal.pone.0073145

**Published:** 2013-09-16

**Authors:** Rajesh K. Sharma, Esma S. Yolcu, Abhishek K. Srivastava, Haval Shirwan

**Affiliations:** Institute for Cellular Therapeutics, Department of Microbiology and Immunology and James Brown Cancer Center, University of Louisville, Louisville, Kentucky, United States of America; New York University, United States of America

## Abstract

The role of CD4^+^ T cells in the generation of therapeutic primary and memory immune responses in cancer diverse immunotherapy settings remains ambiguous. We herein investigated this issue using two vaccine formulations containing a novel costimulatory molecule, SA-4-1BBL, as adjuvant and HPV E7 or survivin (SVN) as tumor associated antigens (TAAs) in two mouse transplantable tumor models; the TC-1 cervical cancer expressing xenogeneic HPV E7 and 3LL lung carcinoma overexpressing autologous SVN. Single vaccination with optimized SA-4-1BBL/TAA formulations resulted in the eradication of 6-day established TC-1 and 3LL tumors in >70% of mice in both models. The *in vivo* depletion of CD4^+^ T cells one day before tumor challenge resulted in compromised vaccine efficacy in both TC-1 (25%) and 3LL (12.5%) tumor models. In marked contrast, depletion of CD4^+^ T cells 5 days post-tumor challenge and one day prior to vaccination did not significantly alter the therapeutic efficacy of these vaccines. However, long-term immunological memory was compromised in the 3LL, but not in TC-1 model as a significant number (85.7%) of tumor free-mice succumbed to tumor growth when rechallenged with 3LL cells 60 days after the initial tumor inoculation. Collectively, these results demonstrate the indispensable role CD4^+^ T cells play in the generation of therapeutic primary immune responses elicited by SA-4-1BBL/TAA-based vaccines irrespective of the nature of TAAs and establish the importance of CD4^+^ T cells for long-term immune memory against 3LL tumor expressing self-antigen SVN, but not TC-1 expressing xenogeneic viral antigen E7.

## Introduction

The critical role of CD4^+^ T cells in the generation of effective and durable adaptive immune responses against various infections, such as intracellular parasites, bacteria, and viruses, has been well documented [1]–[4]. By extrapolation, it is assumed that CD4^+^ T cells also play a similar role in immune responses against tumors, which was substantiated by using autologous tumor-specific CD4^+^ T cells for adaptive immune therapy against cancer [5]. However, the role and relative contribution of CD4^+^ T cells to immune responses generated against cancer *in vivo* in response to active vaccination remains to be fully elucidated. Although CD4^+^T cells have been shown to have direct killing activity against tumor in selected settings [5], it is a common consensus that these cells show their full potential by coordinating effector arms of the immune responses, i.e., helper function. The concept of T cell ‘help’ originated in the 1970 s when it was demonstrated that B cell activation required interactions with CD4^+^ T helper (Th) cells [6]. Subsequent studies have demonstrated the helper function of CD4^+^ T cells for the generation of primary and memory CD8^+^ cytotoxic T lymphocytes (CTLs), which are critical in the elimination of tumors [1]. Natural killer (NK) cells, which are another important effector cell type that has direct killing activity against tumor, have recently been shown to require CD4^+^ T cell help via IL-2 [7].

The chief input of CD4^+^ T cells for anti-tumor effects operates through the direct help for the generation/augmentation of tumor-specific CTL responses [1] and/or indirect help through secretion of various cytokines, such as IL-2 and IFN-γ. IL-2 functions as growth factor for CTLs and is essential for the secondary expansion of memory CD8^+^ T cells [8]. IL-2 can also recruit and retain CTLs at the tumor site. IFN-γ production by CD4^+^ Th1 cells can up regulate MHC molecules on tumor cells, leading to enhanced CTL and Th responses [9]. Recently, it was demonstrated that CD4^+^ T cell help not only promotes CTL expansion in peripheral lymphoid organs, but is also required for the recruitment of low avidity CD8^+^ T cells into tumor microenvironment and augmentation of cytolytic function via up-regulation of granzyme B [10], [11]. Although it is a general consensus that CD4^+^ T cells play an important role in the generation of CD8^+^ T cell responses, these cells may not always require CD4^+^ T cell help. Several studies in various model systems of intrinsic tumor immunity or anti-tumor immunity associated with cancer immunotherapies demonstrated differential requirements for CD4^+^ Th cells during primary CTL responses, thereby resulting in the categorization of CTL responses as Th-dependent or Th-independent [12]–[14]. It has been proposed that high avidity epitopes from foreign antigens may bypass the CD4^+^ T cell help for primary CTL responses due to their ability to induce sufficient IL-2 from CTLs themselves [3], [11], [12], [14]–[17]. Similarly costimulation may play a pivotal role in determining whether or not primary CTL responses will necessitate direct CD4^+^ T cell help [3], [16]–[18]. It has been shown that the agonistic anti-CD40 antibodies or Toll-like receptor (TLR) stimulation can circumvent the need for CD4^+^ T cell help to generate primary and memory CTL responses in some instances [16], [18].

Given the importance of costimulation in the generation of innate and adaptive immune responses and establishment of long-term immune memory [19], we have recently hypothesized the costimulatory member of TNF family ligands as potential adjuvants for therapeutic cancer vaccines and focused on 4-1BBL as the lead candidate [20]–[28,28]. Inasmuch as this molecule functions as cell surface protein and has no function in soluble form [29], we generated a chimeric recombinant 4-1BBL where the extracellular portion of this molecule was cloned C-terminus to core streptavidin (SA-4-1BBL) [30]. This chimeric molecule exists as tetramers/oligomers and has potent costimulatory activity on CD4^+^ and CD8^+^ T cells in soluble form [21], [30]. A single vaccination with SA-4-1BBL as adjuvant component of E7 or SVN as TAA had therapeutic efficacy in eliminating the majority of tumors expressing these TAAs in mice [24]. The therapeutic efficacy of vaccines was associated with heightened CTL and NK cellular and IL-2 and IFN-γ cytokine responses [25], [28].

We herein optimized the dose of vaccine formulations, tested the therapeutic efficacy of multiple vaccinations in the TC-1 model, and investigated the role of CD4^+^ T cells in vaccine generated therapeutic primary and long-term immune memory responses. Multiple vaccinations with the optimized vaccine formulation resulted in 100% therapeutic efficacy. Importantly, the depletion of CD4^+^ T cells before tumor inoculation compromised vaccine efficacy in both 3LL and TC-1 tumor models, while depletion one day before vaccination in mice with established tumors had no significant effect on the therapeutic efficacy of the vaccines. However, mice that underwent successful immunotherapy for SVN expressing 3LL, but not E7 expressing TC-1, failed to control tumor growth when subsequently challenged with the same tumor cells, demonstrating defective immune memory. Taken together, these studies demonstrate that CD4^+^ T cell help is critical for the generation of primary therapeutic immune responses and that the help for the generation of long-term memory may depend on the nature of antigen used for vaccination.

## Results

### Optimization of SA-4-1BBL/E7 vaccine formulations for the most effective therapy in the TC-1 tumor model

**Figure 1:**
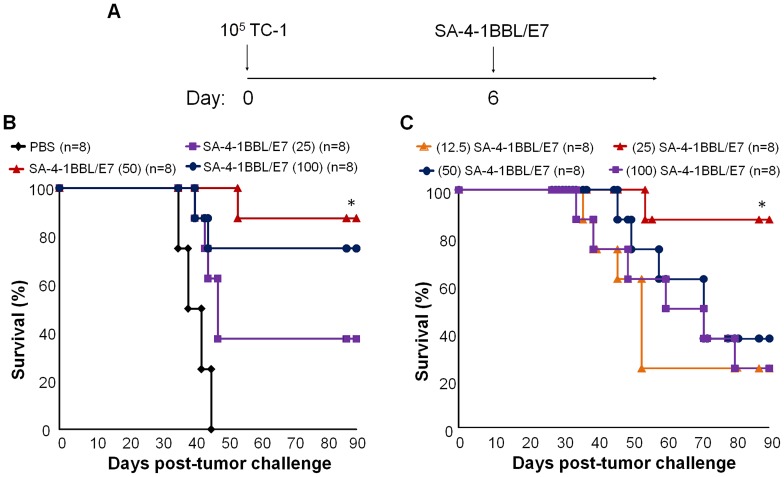
Optimizing the SA-4-1BBL/E7 vaccine formulation for the most effective therapy in the TC-1 cervical cancer mouse model. C57BL/6 mice were inoculated s.c. with 1×10^5^ live TC-1 tumor cells and vaccinated with the indicated doses of E7 and SA-4-1BBL proteins on day 6 post-tumor challenge. Mice vaccinated with PBS were used as controls. (A) Schematic depiction of the experimental procedure. (B) Optimizing the dose of E7 antigen. Mice were vaccinated with the indicated doses of E7 in µg with a fixed amount of SA-4-1BBL (25 μg). *P<0.05 vs. all other groups, except E7 100 µg. (C) Optimizing the dose of SA-4-1BBL. Mice were vaccinated with the indicated doses of SA-4-1BBL with the optimum 50 µg dose of E7 as determined in (B). The 25-μg SA-4-1BBL treatment group was significant (*P<0.05) from all other groups and found to be the optimal dose. The SA-4-1BLL/E7 (25 /50 µg) group from panel A is re-graphed for direct comparison with the other groups.

We have previously demonstrated that single immunization with a vaccine formulation containing 25 µg of SA-4-1BBL and 50 µg of E7 protein had ∼70% efficacy in the established TC-1 tumor model [25]. Therefore, we herein tested if this therapeutic efficacy can further be improved by optimizing the dose of SA-4-1BBL and E7 component of the vaccine formulation. C57BL/6 mice were challenged with 1×10^5^ live TC-1 tumor cells s.c. on the right flank and then vaccinated s.c. once on day 6 post tumor challenge with various vaccine formulations(Fig. 1A). Inasmuch as in previous studies we demonstrated 25 µg of SA-4-1BBL having therapeutic efficacy in various settings [24]–[26], [28], this dose was tested in combination with various doses (25, 50, and 100 µg) of the E7 protein. Vaccination with 50 μg of E7 had the most therapeutic efficacy as ∼87% of mice had tumor-free survival over the 90-day observation period (Fig. 1B). Vaccine formulation containing 100 μg of E7 protein did not enhance tumor-free survival, and if anything, there was slight reduction in therapeutic efficacy as compared with the 50 µg dose. Vaccination with a formulation containing 25 µg of E7 resulted in sub-optimal efficacy with 37.5% tumor-free survival (Fig. 1B).

**Figure 2:**
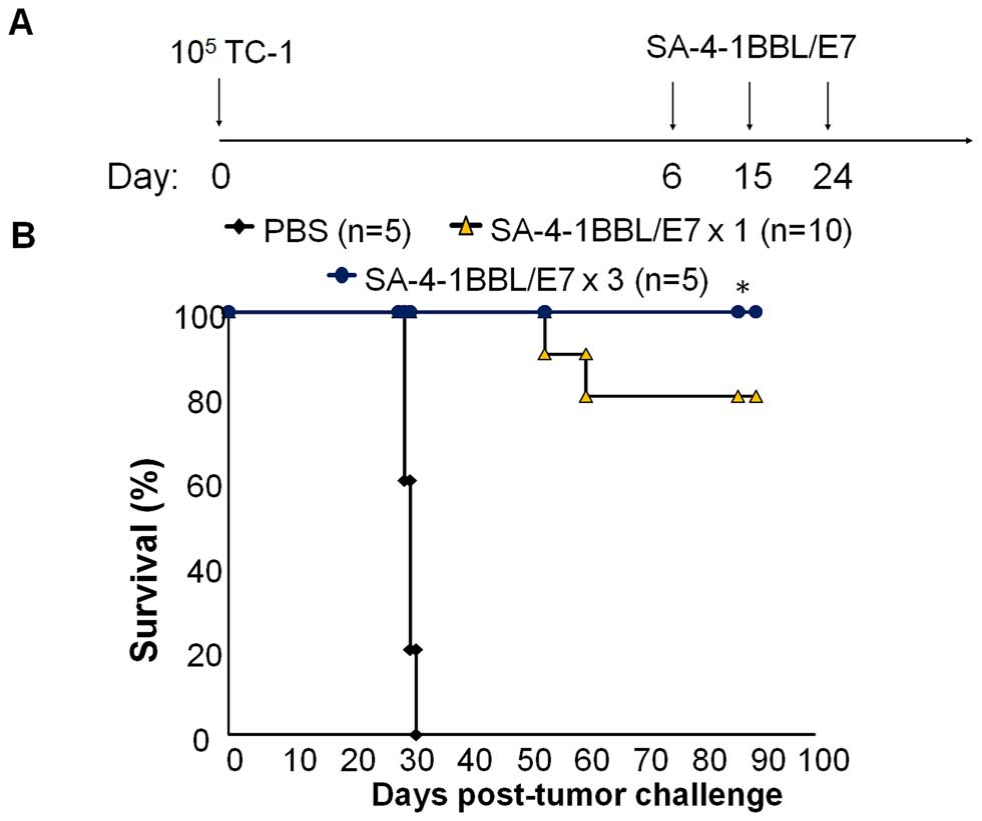
The therapeutic efficacy of SA-4-1BBL/E7 vaccine is improved by multiple vaccinations in the TC-1 cervical cancer model. (A) Schematic depiction of the experimental procedure. (B) C57BL/6 mice were immunized s.c. with the optimized SA-4-1BBL/E7 (25 µg/50 µg) vaccine formulation on day 6 post-challenge with 1×10^5^ live TC-1 tumor cells. A group of mice received two more vaccinations administered on days 15 and 24 post-tumor challenge. Mice vaccinated with PBS were used as controls. P<0.05 *vs.* PBS group, but not SA-4-1BBL/E7 x 1 group.

We next used the 50-µg dose of E7 with varying amounts (12.5, 25, 50, 100 µg) of SA-4-1BBL to optimize the dose of adjuvant. Vaccination with 25 µg of SA-4-1BBL had the most therapeutic efficacy as compared with all the other doses (Fig. 1C). Interestingly, higher doses of SA-4-1BBL significantly compromised the efficacy of the vaccine. This observation is consistent with a study using higher doses of agonistic Abs against 4-1BB receptor [31]. We next tested if multiple vaccinations with the optimized SA-4-1BBL/E7 vaccine formulation improve its therapeutic efficacy over a single vaccination. All the mice vaccinated with SA-4-1BBL/E7 (25/50 µg) formulation three times on day 6, 15, 24 post-tumor inoculation with live 1×10^5^ live TC-1 cells (Fig. 2A) achieved tumor-free survival for the 90-day observation period (Fig. 2B).

### Immune responses generated by the SA-4-1BBL/E7 vaccine formulation are systemic, long-lasting, and tumor type specific

**Figure 3:**
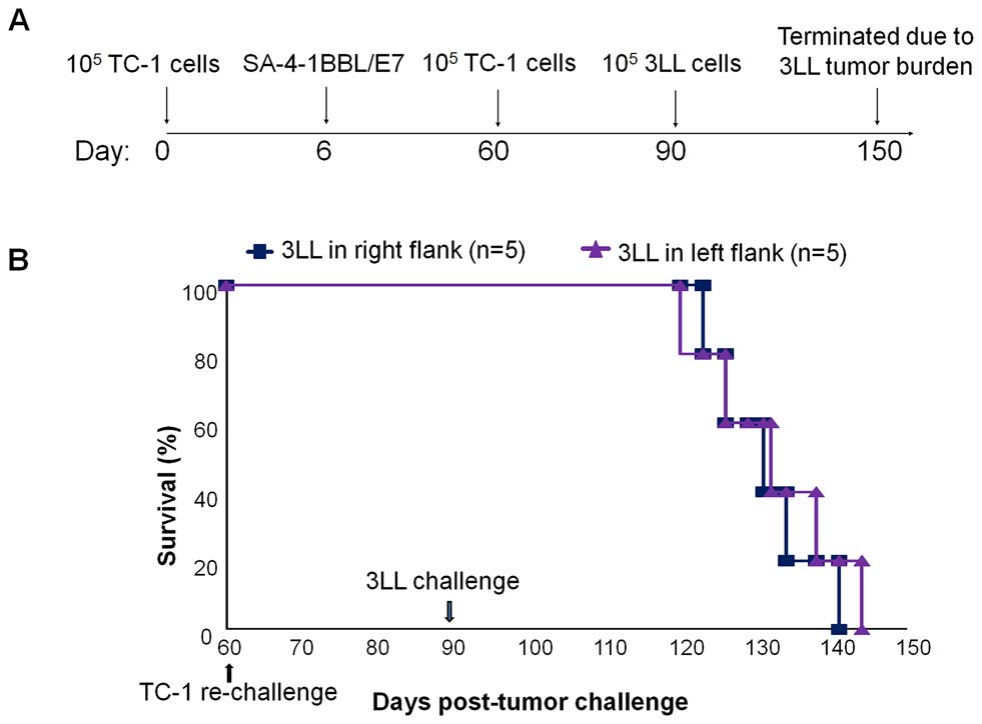
Long-term immune memory induced by SA-4-1BBL/E7 vaccine is systemic and tumor-specific. C57BL/6 mice that had eradicated TC-1 tumors in response to vaccination with SA-4-1BBL/E7 were given a second dose of TC-1 tumor cells (1×10^5^) in either left or right flank 60 days after primary tumor challenge to evaluate long-term immune memory and if the memory is systemic. (A) Schematic depiction of the experimental procedure. (B) Survival. None of the mice developed TC-1 tumors in left or right flanks within the 30-day observation period at which time the animals were inoculated with live 1×10^5^ heterologous 3LL tumor cells into the opposite flanks to the last TC-1 challenge. All long-term mice developed 3LL tumors in a similar tempo to naïve (data not shown) mice without recurrence of TC-1 tumor growth, demonstrating that anti-tumor immune responses are systemic and tumor type-specific.

We have previously reported that therapeutic vaccination with SA-4-1BBL/E7 resulted in long-term immune memory that protected mice against a second challenge with live TC-1 tumor cells injected s.c. in the same flank as the first inoculation [25]. We herein investigated if the long-term immune memory achieved by the vaccine is localized or systemic and tumor type-specific. Mice that eradicated tumors in response to SA-4-1BBL/E7 vaccination were challenged with live TC-1 cells either on the same flank as the first inoculation 60 days earlier or on the opposite flank (Fig. 3A). As we have previously reported [24], [25], [27], challenge with 1×10^5^ TC-1 live tumor cells results in palpable tumors within 15 days. None of the mice developed tumor following post-secondary challenge in these long term vaccinated mice for an additional 30 days, demonstrating that the vaccine-generated immune memory was systemic (Fig. 3B). The vaccine generated long-term immune memory was tumor type specific as all the long-term mice that remained tumor-free post-secondary inoculation with TC-1 cells did not control the growth of the heterologous 3LL cells (Fig. 3B). Importantly, none of the mice expired form 3LL tumor burden had TC-1 recurrence.

### Depletion of CD4^+^ T cells post-tumor challenge and before vaccination with SA-4-1BBL/TAAs has no effect on the generation of therapeutic immune responses against TC-1 and 3LL tumors

**Figure 4:**
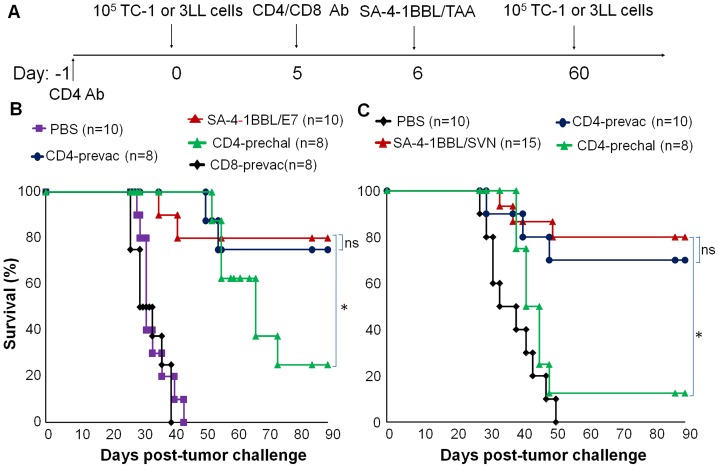
CD4^+^ T cells play a critical role in the SA-4-1BBL/TAAs-induced therapeutic primary responses against tumors. (A) Schematic depiction of the experimental procedure. (B) Assessing the role of CD4+ and CD8+ T cells in the SA-4-1BBL/E7 vaccine induced therapeutic immunity against TC-1 tumor. C57BL/6 mice were treated i.p. with depleting doses of Abs against CD4 (500 μg/mouse) or CD8 (500 μg/mouse) either one day before vaccination (CD4 or CD8-prevac) with an optimized formulation of SA-4-1BBL/E7 (25/50 µg) administered on day 6 post-tumor challenge or one day before tumor challenge (CD4-prechal) Controls included mice without T cell depletion vaccinated with SA-4-1BBL/E7 or PBS. (C) Assessing the role of CD4+ T cells in the SA-4-1BBL/SVN vaccine induced therapeutic immunity against 3LL tumor. C57BL/6 mice were inoculated with 1×10^5^ 3LL tumor cells s.c. and vaccinated with SA-4-1BBL/SVN (25/50 µg) on day 6 post-tumor challenge. One group of mice received the depleting dose of anti-CD4 Ab one day before tumor challenge (CD4-prechal) while another group received the Ab one day before vaccination (CD4-prevacc) *P<0.01; ns, not significant. Some of the mice in SA-4-1BBL/SVN and CD4-prevac groups have recently been published (ref. 31) and pooled with more mice and graphed for comparison purposes.

We have previously shown that the therapeutic efficacy of SA-4-1BBL-based vaccines is associated with augmented CD4^+^ and CD8^+^ T cells responses [25]. However, the relative role of these two cell types in therapeutic efficacy elicited by the SA-4-1BBL/E7 vaccine formulation remains to be elucidated. To provide direct evidence and assess the relative roles of these cells, we herein used antibodies to CD4 and CD8 molecules to deplete CD4^+^ or CD8^+^ T cells one day before vaccination with the optimized therapeutic SA-4-1BBL/E7 formulation using the TC-1 tumor model (Fig. 4A). Depletion of CD8^+^ T cells one day before vaccination completely abolished therapeutic efficacy, whereas depletion of CD4^+^ T cells had no significant effect on vaccine efficacy (Fig. 4B).

Inasmuch as therapeutic immune responses in the TC-1 model are dictated by CD8^+^ T cells against a dominant E7 epitope [24], we asked the question if CD4^+^ T cell response is required for therapeutic efficacy of vaccines using weak autologous TAA. The depletion of CD4^+^ T cells one day before vaccination with SA-4-1BBL admixed with survivin (SVN) as self TAA had no detectable effect on the therapeutic efficacy of the vaccine in the 3LL lung carcinoma model expressing SVN (Fig. 4C). Taken together, these data demonstrate that CD4^+^ T cell help is not required for the therapeutic immune responses generated by SA-4-1BBL-based vaccination and the lack of CD4^+^ T cell role is independent of TAAs and tumor models used in this study.

### Depletion of CD4^+^ T cells pre-tumor challenge compromises the therapeutic efficacy SA-4-1BBL/TAA vaccines against TC-1 and 3LL tumors

The lack of a positive effect of CD4^+^ T cells on the therapeutic efficacy of SA-4-1BBL-based vaccines in the 3LL and TC-1 models as demonstrated above does not necessarily rule out the contribution of these cells in vaccine generated responses. CD4^+^ T cells may be required for the initiation of immune responses, but not necessarily perpetuation/maintenance of such responses. In support of this notion, we found that the depletion of CD4^+^ T cells one day before TC-1 tumor challenge dramatically compromised the efficacy of SA-4-1BBL/E7 vaccine as 75% mice developed tumors and succumbed to death as compared with ∼20% mice in the group without depletion. The importance of CD4^+^ T cells in the therapeutic efficacy of SA-4-1BBL/SVN vaccine was also demonstrated as 88% mice succumbed to tumor growth after depletion of CD4^+^ T cells one day before inoculation with 3LL tumor cells as compared with 20% mice in the non-depleted control group (Fig. 4C).

### CD4^+^ T cells are required for the long-term immune memory established by vaccination with SA-4-1BBL and survivin as self TAA, but not E7 as xenogeneic viral TAA

**Figure 5:**
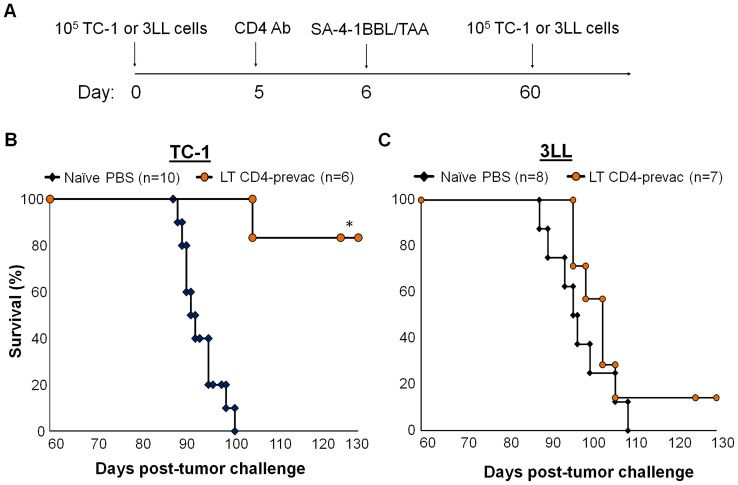
CD4^+^ T cells are required for SA-4-1BBL/TAAs vaccine-induced long-term immune memory against 3LL, but not against TC-1 tumor. (A) Schematic depiction of the experimental procedure. (B) Long-term C57BL/6 mice that had eradicated TC-1 tumors irrespective of CD4+ T cell depletion one day before vaccination with SA-4-1BBL/E7 had long-term protective immune response as assessed by a secondary challenge with TC-1 tumor cells 60 days after the initial tumor inoculation. Naïve mice injected with PBS and challenged with 1×10^5^ TC-1 cells served as controls and expired from tumor burden within 40 days. (C) Long-term C57BL/6 mice that had eradicated 3LL tumors irrespective of CD4+ T cell depletion one day before vaccination with SA-4-1BBL/SVN had significantly compromised memory immune response as assessed by a secondary challenge with 3LL tumor cells 60 days after the initial tumor inoculation. Naïve mice injected with PBS and challenged with 1×10^5^ 3LL cells served as controls and expired from tumor burden within 50 days.

Since depletion of CD4^+^ T cells one day prior to vaccination did not alter the vaccine efficacy in both TC-1 and 3LL tumor models, we sought to investigated if the absence of CD4^+^ T cells during the primary phase of immune response have any effect on the establishment of long term immunological memory. Toward this end, mice that had eradicated primary tumors in response to vaccination one day post-CD4^+^ T cell depletion were given a second dose of tumor cells 60 days post primary tumor challenge (Fig. 5A). While only 1/6 mice developed tumor following secondary tumor challenge in the TC-1 cohort vaccinated with SA-4-1BBL/E7 (Fig. 5B), 6/7 mice in the 3LL group vaccinated with SA-4-1BBL/SVN developed tumor following the secondary tumor challenge (Fig. 5C). Taken together, these data demonstrate that CD4^+^ T cells are critical to the establishment of SA-4-1BBL-mediated long-term immune memory to self, potentially weak TAAs, but not xenogeneic strong TAAs.

## Discussion

The importance of CD4^+^ T cells in the establishment of primary and memory immune responses to pathogens is well established [2]–[4], [12], [16]. However, the role of CD4^+^ T cells in anti-tumor immune responses generated by various immunotherapeutic strategies, particularly active vaccination, remains to be fully elucidated. In the present study, we demonstrated that: i) primary therapeutic immune responses against both the TC-1 cells expressing a strong xenogeneic TAA E7 and 3LL expressing a weak self TAA SVN require CD8^+^ T cells; ii) CD4^+^ T cell help is essential during the priming phase for the therapeutic efficacy of vaccines as the elimination of CD4^+^ T cells before tumor challenge resulted in vaccine failure, irrespective of TAAs used for vaccination; iii) depletion of CD4^+^ T cells following priming phase by the tumor cells, but one day before vaccination, did not negatively impact vaccine therapeutic efficacy against the tumors in both models; and iv) the depletion of CD4^+^ T cells one day before vaccination resulted in the failure of protective recall responses generated by SVN as a weak self TAA, but not E7 as a strong xenogeneic viral TAA.

Tumor growth and progression are the end result of coordinated molecular and cellular interactions that circumvent immunosurveillance mechanisms. Therefore, immunotherapy has great potential to serve as an effective and less toxic alternative to current standard treatments for cancer. Subunit vaccines based on TAAs represent an attractive immunotherapeutic approach for the treatment of cancer. However, the success of TAA-based vaccines hinges upon their ability to generate not only robust immune effector responses, but also overcome various immune evasion mechanisms employed by the progressing tumor. As such, TAA-based vaccines may benefit from adjuvants having pleiotropic effects on various immune cells that are critical for tumor eradication. We have recently generated a novel from of 4-1BBL chimeric with streptavidin (SA-4-1BBL) and demonstrated its robust costimulatory activity on CD4^+^ T and CD8^+^ T cells, which translated into therapeutic efficacy in various tumor models when SA-4-1BBL was used as adjuvant component of TAA-based vaccines [24], [25], [28], [30]. In this study, we optimized the formulation of SA-4-1BBL/E7 vaccine and demonstrated that a single vaccination was effective in eradicating established E7 expressing TC-1 tumors with 70-87.5% efficacy, which was further improved to 100% by multiple vaccinations. Similarly, we have recently reported that vaccination with SA-4-1BBL/SVN in a prime-boost setting is also effective in eradicating the 3LL tumor in 100% of mice [28].

CD8^+^ T cells play a requisite role for the therapeutic efficacy of SA-4-1BBL-based vaccines in both tumor models. Immune responses to E7 expressing TC-1 tumors are driven by CD8^+^ T cells responding to a dominant E7 epitope (E7_49–57_). Immunization with a synthetic peptide representing this epitope has shown therapeutic efficacy against HPV-16 E7 expressing tumors [24], providing indirect evidence for the critical role of CD8^+^ T cells. As a direct evidence, physical depletion of CD8^+^ T cells results in the abrogation of vaccine efficacy as demonstrated in this study and by others [32]. The therapeutic efficacy of SA-4-1BBL/SVN vaccine also requires CD8^+^ T cells as effector arm as physical depletion of these cells resulted in complete failure of vaccine in the 3LL tumor model [28].

Similarly, depletion of CD4^+^ T cells before tumor challenge significantly negated therapeutic efficacy of the vaccines in both tumor models. This negative effect was pronounced more in the 3LL as compared with the TC-1 model (12.5 vs 25% survived). This may be due to the effect of stimulation of CD8^+^ T cells with a strong xenogeneic TAA, E7, in the case of TC-1 model as compared with a weak self-antigen SVN in the 3LL model. Given that CD8^+^ T cells play a critical role for the elimination of tumor in both models, we speculate that the effect of CD4^+^ T cell depletion on therapeutic efficacy of vaccines goes through CD8^+^ T cells as a downstream target. In this context, our data is consistent with studies reporting that primary CD8^+^ T cell responses against noninflammatory antigens, such as transplantation antigens, soluble antigens, require CD4^+^ T cell help [33], [34]. Although it was initially thought that CD4^+^ T cell help is not required for the generation of CD8^+^ T cell primary responses against infections due to the ability of infections to provide inflammatory signals for full activation of DCs to bypass the CD4^+^ T cell help, it is now clear that certain infections, such as vaccinia virus, influenza virus, adenovirus, also require CD4^+^ T cell help for CD8^+^ T cell priming [35]–[37]. Although we do not know the mechanistic basis of our observation related to the requirement for CD4^+^ T cell help for the generation of therapeutic primary immune responses against cancer, it is tempting to speculate that CD4^+^ T cells may facilitate vaccine efficacy by several means. First, CD4^+^ T cells primed by the tumor cells may get activated, upregulate 4-1BB on their surface, and become the direct target of SA-4-1BBL following vaccination for i) licensing DCs through CD40-CD40L interaction, ii) generation of cytokines that help CD8^+^ T cells for expansion and effector differentiation, and iii) providing survival benefits by regulating extrinsic, such as TRAIL, and/or intrinsic, such as Bcl-x_L_, apoptotic mechanisms [38]. In particular, licensed DCs is expected to upregulate costimulatory molecules, particularly 4-1BB, which in turn may serve as the target for SA-4-1BBL for antigen uptake and cross-presentation for the generation of effective CD8^+^ T cell responses against tumor, which we have recently reported [26]. Second, intratumoral CD4^+^ T cells were shown to recruit CD8^+^ T cells, enhance their proliferation, survival, and cytolytic function within the tumor microenvironment [39]. Third, CD4^+^ T cells may also help vaccine primary therapeutic efficacy through their effect on various innate immune cells, such as macrophages and NK cells and/or elaboration of cytokines, such as IFN-γ. In particular, IFN-γ has been shown to directly act on CD8^+^ T cells to increase their abundance in an acute viral infection model [40].

In marked contrast, physical elimination of CD4^+^ T cells one day before vaccination had no effect on the therapeutic efficacy in both tumor models. We interpret this observation to mean that the requirement for CD4^+^ T cell help for vaccines therapeutic efficacy, irrespective of its mechanistic nature, has already been fulfilled by priming with the tumor. The presence of ongoing immune responses to tumors, irrespective of tumor progression, has been demonstrated in various preclinical and clinical settings [24], [41]. Therefore, immunization with SA-4-1BBL/E7 or SA-4-1BBL/SVN vaccine formulations simply capitalizes on the preexisting immunity and drives such immune responses to a therapeutic scale.

We have recently demonstrated that vaccination with SA-4-1BBL/TAAs results in TAA-specific CD4^+^ and CD8^+^ T cell primary and long-term memory responses with therapeutic efficacy [25]. Mice with effective immunotherapy controlled recurrences when challenged with the original tumor in both 3LL and TC-1 models, demonstrating the existence of functional long-term immunological memory [24], [25], [28]. The long-term memory response was further substantiated by increased memory pool for both CD4^+^ and CD8^+^ T cells and enhanced T cell proliferative, killing, and Th1 cytokine responses in long-term surviving mice with effective therapy [24], [25], [28]. We herein demonstrated that the long-term memory is systemic and tumor specific as mice that had eradicated primary TC-1 tumors succumb to tumor growth when rechallenged with a secondary dose of 3LL tumor cells, but not TC-1 tumors. Given the critical role of CD8^+^ T cells in recall responses against tumor and control of recurrences and the demonstrated role of CD4^+^ T cells in the generation/maintenance of CD8^+^ T cell memory responses under selected settings, we assessed the effect of CD4^+^ T cells depletion during the primary responses on long-term immune memory against TC-1 expressing xenogeneic viral E7 TAA vs. 3LL expressing autologous self-antigen SVN. Interestingly, only 1/6 long-term tumor-free mice, depleted for CD4^+^ T cells one day before vaccination, in the TC-1 model developed tumor when challenged with the secondary dose of TC-1 cells 60 days post-primary tumor challenge. In marked contrast, 6/7 mice in the 3LL model succumbed to tumor growth upon the secondary challenge with the 3LL cells, suggesting a profound defect in establishment/persistence and functionality of memory responses.

These observations are consistent with findings in various models showing that the burst size and quality of primary CD8^+^ T cell responses determine if CD4^+^ T cell help is required. Immunization with dominant epitopes generates CD8^+^ T cell memory responses that do not require CD4^+^ T cell help, whereas immunization with subdominant epitopes does [3], [11], [12], [14]–[17]. However, we do not know the exact mechanistic basis of observations in our model. HPV E7 contains a dominant CD8^+^ T cell epitope and vaccination with a synthetic peptide representing this epitope generates effective primary as well as recall CD8^+^ T cell responses against TC-1 tumor as demonstrated by us and others [24], [25], [42]. It is tempting to speculate that challenge with the TC-1 tumor expressing E7 TAA primes both CD4^+^ and CD8^+^ T cells to this TAA before vaccination. During this priming phase by the tumor, CD8^+^ T cells are instructed by CD4^+^ T cells to generate memory recall responses, and as such their elimination one day before vaccination has a null effect on the generation of recall responses. Vaccination with SA-4-1BBL/E7 may simply expand the already primed CD8^+^ T cells “clonal burst” by providing high MHC/peptide density and/or strong costimulation by SA-4-1BBL or other costimulatory molecules [43], [44], resulting in the generation of strong primary and recall responses. In marked contrast, challenge with 3LL tumor cells expressing survivin as a weak TAA may not results in effective priming of CD8^+^ T cells, survival, and clonal burst, and as such require CD4^+^ T cell help not only during priming with tumor, but also vaccination with SA-4-1BBL/SVN to expand SVN-primed CD8^+^ T cells and/or affect the quality of these cells to the levels that are required for the generation of protective CD8^+^ T cell recall responses. Removal of CD4^+^ T cells one day before vaccination, therefore, may limit the clonal burst/quality of survivin-primed CD8^+^ T cells by various means; i) limiting IL-2 elaboration required for CD8^+^ T cell expansion and survival; ii) lack of direct expansion of CD8^+^ T cells via CD40L/CD40 interaction; iii) lack of help for survival either by downregulating TRAIL and/or upregulating Bcl-x_L_; and iv) potential other means. Further studies are required to assess the relative contribution of the enumerated mechanisms on the observed outcomes, and particularly if the observed defect(s) in the CD8^+^ T cell recall responses against the 3LL tumor are quantitative or qualitative.

Taken together, these results demonstrate that vaccination with SA-4-1BBL/TAA is effective in generating primary and long-term systemic and tumor-specific immune responses with therapeutic efficacy against 3LL carcinoma and TC-1 cervical cancer models. CD8^+^ T cells play an obligatory role for vaccine therapeutic efficacy in both models as demonstrated here (Fig. 4A) and by a previously published study [28]. Importantly, CD4^+^ T cells were required for the generation of therapeutic primary immune responses for both tumor models, but the requirement for CD4^+^ T cell help for the generation/maintenance of long-term immune memory was dependent on the nature of TAA, SVN as self TAA required CD4^+^ T cells, whereas HPV E7 as xenogeneic viral TAA bypassed such a requirement.

## Materials and Methods

### Mice and cell lines

C57BL/6 mice (6–8 wk old) were purchased from The Jackson Laboratory or bred in our animal facility at the University of Louisville. All animals were cared for in accordance with institutional and National Institute of Health guidelines. TC-1 cell line was purchased from ATTC and 3LL cell line was a gift from late RD Stout that was originally purchased from ATCC and maintained as previously reported [24]–[26], [28].

### Reagents

Fluorochrome-conjugated Abs (anti-CD4-APC, anti-CD8-PerCP, anti-CD3 and SA-PerCP) and isotype controls were purchased from BD Pharmingen and eBioscience. Unlabeled depleting antibodies to mouse CD4 (GK1.5) and CD8 (53.6.72) were purchased from Bio X Cell (West Lebanon, NH). Recombinant HPV16 E7, mouse survivin, and SA-4-1BBL proteins were produced as published previously [24]–[26]. All the proteins had minimal endotoxin levels; E7 (0.021 EU/μg), survivin (0.066 EU/μg), and SA-4-1BBL (0.004 EU/μg).

### Tumor models and vaccination

For therapeutic studies, C57BL/6 mice were inoculated s.c. into the right back flank with live 1×10^5^ TC-1 or 3LL tumor cells in 200 µl of PBS and immunized s.c. on day 6 post-tumor challenge or as mentioned in results and figure legends with various vaccine formulations containing SA-4-1BBL as adjuvant and E7 or survivin as TAAs. Mice with PBS vaccination were used as controls. Tumor growth was monitored 2–3 times per week and tumor size was measured in mm using a caliper. Average tumor size was calculated by measuring two perpendicular diameters. The survival end-point was defined as tumor size of 12 mm in diameter, grade 3 tumor ulceration, or when mice showed signs of poor conditions (hunched posture, anorexia, dehydration, weight loss, inactivity, difficulty walking, dyspnea, or moribund).

To assess the SA-4-1BBL/TAA vaccine-induced immune memory response against tumors, long-term survivors were given a second dose of live 1×10^5^ TC-1 or 3LL tumor cells 60 days after the primary tumor challenge. These mice were then monitored for tumor development for an additional 30 days. In the TC-1 model, mice that had eradicated tumor in response to vaccination were given a second dose of live TC-1 tumor cells in either left or right flank 60 days post-primary tumor challenge to evaluate localized *vs.* systemic antitumor immune responses. These mice were monitored for 30 days without detectable tumor growth and then challenged with a live dose (1×10^5^) of heterologous 3LL tumor cells in opposite flanks to TC-1 cell inoculation to evaluate the tumor specific immune memory responses.

### T cell depletion

The depletion of CD4^+^ T cells was performed in two different settings. In the first setting, C57BL/6 challenged with live 1×10^5^ TC-1 or 3LL cells were given an injection of Ab against mouse CD4 molecule (500 µg/mouse, clone GK1.5) 5 days after tumor inoculation and one day before vaccination with SA-4-1BBL/TAAs. In this setting, an Ab to mouse CD8 molecule (clone 53.6.72) was also given (500 µg/mouse) to deplete CD8^+^ T cells in the TC-1 tumor model. In the second setting, the CD4^+^ T cell depletion was performed one day before tumor inoculation. The depletion of individual cell type was assessed 5 days post Ab-treatment using flow cytometry and was found to be almost complete (>98%).

### Statistical analysis

Statistical analysis was done using the long-rank test for comparing the survival results among the various groups for obtaining the p values in SPSS software. For each test, *p* value less than 0.05 and 0.001 were considered significant (*) and very significant (**), respectively.

### Ethics statement

This study was approved by the University of Louisville Institutional Animal Care and Use Committee (IACUC) (Protocol number: 08142) and carried out in strict accordance with the recommendations in the Guide for the Care and Use of Laboratory Animals of the National Institutes of Health.

## References

[1] Keene JA, Forman J (1982) Helper activity is required for the in vivo generation of cytotoxic T lymphocytes. J Exp Med 155: 768–782. 6801178 10.1084/jem.155.3.768 PMC2186611

[2] Sun JC, Bevan MJ (2003) Defective CD8 T cell memory following acute infection without CD4 T cell help. Science 300: 339–342. 12690202 10.1126/science.1083317 PMC2778341

[3] Sun JC, Williams MA, Bevan MJ (2004) CD4+ T cells are required for the maintenance, not programming, of memory CD8+ T cells after acute infection. Nat Immunol 5: 927–933. 15300249 10.1038/ni1105 PMC2776074

[4] Swain SL, McKinstry KK, Strutt TM (2012) Expanding roles for CD4(+) T cells in immunity to viruses. Nat Rev Immunol 12: 136–148. 22266691 10.1038/nri3152 PMC3764486

[5] Hirschhorn-Cymerman D, Budhu S, Kitano S, Liu C, Zhao F, et al (2012) Induction of tumoricidal function in CD4+ T cells is associated with concomitant memory and terminally differentiated phenotype. J Exp Med 209: 2113–2126. 23008334 10.1084/jem.20120532 PMC3478933

[6] Mitchison NA (1971) The carrier effect in the secondary response to hapten-protein conjugates. II. Cellular cooperation. Eur J Immunol 1: 18–27. 14978857 10.1002/eji.1830010104

[7] van den Broeke LT, Daschbach E, Thomas EK, Andringa G, Berzofsky JA (2003) Dendritic cell-induced activation of adaptive and innate antitumor immunity. J Immunol 171: 5842–5852. 14634094 10.4049/jimmunol.171.11.5842

[8] Williams MA, Tyznik AJ, Bevan MJ (2006) Interleukin-2 signals during priming are required for secondary expansion of CD8+ memory T cells. Nature 441: 890–893. 16778891 10.1038/nature04790 PMC2776073

[9] Weidanz JA, Nguyen T, Woodburn T, Neethling FA, Chiriva-Internati M, et al (2006) Levels of specific peptide-HLA class I complex predicts tumor cell susceptibility to CTL killing. J Immunol 177: 5088–5097. 17015692 10.4049/jimmunol.177.8.5088

[10] Bos R, Sherman LA (2010) CD4+ T-cell help in the tumor milieu is required for recruitment and cytolytic function of CD8+ T lymphocytes. Cancer Res 70: 8368–8377. 20940398 10.1158/0008-5472.CAN-10-1322 PMC2970736

[11] Wong SB, Bos R, Sherman LA (2008) Tumor-specific CD4+ T cells render the tumor environment permissive for infiltration by low-avidity CD8+ T cells. J Immunol 180: 3122–3131. 18292535 10.4049/jimmunol.180.5.3122

[12] Janssen EM, Lemmens EE, Wolfe T, Christen U, von Herrath MG, et al (2003) CD4+ T cells are required for secondary expansion and memory in CD8+ T lymphocytes. Nature 421: 852–856. 12594515 10.1038/nature01441

[13] Mintern JD, Davey GM, Belz GT, Carbone FR, Heath WR (2002) Cutting edge: precursor frequency affects the helper dependence of cytotoxic T cells. J Immunol 168: 977–980. 11801627 10.4049/jimmunol.168.3.977

[14] Wang JC, Livingstone AM (2003) Cutting edge: CD4+ T cell help can be essential for primary CD8+ T cell responses in vivo. J Immunol 171: 6339–6343. 14662830 10.4049/jimmunol.171.12.6339

[15] Lin CT, Chang TC, Shaw SW, Cheng PJ, Huang CT, et al (2006) Maintenance of CD8 effector T cells by CD4 helper T cells eradicates growing tumors and promotes long-term tumor immunity. Vaccine 24: 6199–6207. 16824651 10.1016/j.vaccine.2006.05.108

[16] Schoenberger SP, Toes RE, van der Voort EI, Offringa R, Melief CJ (1998) T-cell help for cytotoxic T lymphocytes is mediated by CD40-CD40L interactions. Nature 393: 480–483. 9624005 10.1038/31002

[17] Velders MP, Markiewicz MA, Eiben GL, Kast WM (2003) CD4+ T cell matters in tumor immunity. Int Rev Immunol 22: 113–140. 12962272 10.1080/08830180305220

[18] Bullock TN, Yagita H (2005) Induction of CD70 on dendritic cells through CD40 or TLR stimulation contributes to the development of CD8+ T cell responses in the absence of CD4+ T cells. J Immunol 174: 710–717. 15634890 10.4049/jimmunol.174.2.710

[19] Croft M (2003) Co-stimulatory members of the TNFR family: keys to effective T-cell immunity? Nat Rev Immunol 3: 609–620. 12974476 10.1038/nri1148

[20] Schabowsky RH, Sharma RK, Madireddi S, Srivastava A, Yolcu ES, et al (2009) ProtEx technology for the generation of novel therapeutic cancer vaccines. Exp Mol Pathol 86: 198–207. 19454266 10.1016/j.yexmp.2009.01.010 PMC2917214

[21] Schabowsky RH, Elpek KG, Madireddi S, Sharma RK, Yolcu ES, et al (2009) A novel form of 4-1BBL has better immunomodulatory activity than an agonistic anti-4-1BB Ab without Ab-associated severe toxicity. Vaccine 28: 512–522. 19836479 10.1016/j.vaccine.2009.09.127 PMC2805442

[22] Singh NP, Yolcu ES, Taylor DD, Gercel-Taylor C, Metzinger DS, et al (2003) A novel approach to cancer immunotherapy: tumor cells decorated with CD80 generate effective antitumor immunity. Cancer Res 63: 4067–4073. 12874008

[23] Singh NP, Miller RW, Yolcu ES, Kilinc MO, Oechsli M, et al (2006) Primary tumor cells resected from cancer patients and decorated with a novel form of CD80 protein serve as effective antigen-presenting cells for the induction of autologous T cell immune responses ex vivo. Hum Gene Ther 17: 334–346. 16544982 10.1089/hum.2006.17.334

[24] Sharma RK, Elpek KG, Yolcu ES, Schabowsky R-H, Zhao H, et al (2009) Costimulation as a platform for the development of vaccines: a peptide-based vaccine containing a novel from of 4-1BBL eradicates established tumors. Cancer Res 69: 4319–4326. 19435920 10.1158/0008-5472.CAN-08-3141 PMC2755220

[25] Sharma RK, Srivastava AK, Yolcu ES, MacLeod KJ, Schabowsky RH, et al (2010) SA-4-1BBL as the immunomodulatory component of a HPV-16 E7 protein based vaccine shows robust therapeutic efficacy in a mouse cervical cancer model. Vaccine 28: 5794–5802. 20603135 10.1016/j.vaccine.2010.06.073 PMC2921468

[26] Sharma RK, Schabowsky R-H, Srivastava A, Elpek KG, Madireddi S, et al (2010) 4-1BB ligand as an effective multifunctional immunomodulator and antigen delivery vehicle for the development of therapeutic cancer vaccines. Cancer Res 70: 3945–3954. 20406989 10.1158/0008-5472.CAN-09-4480 PMC2872136

[27] Sharma RK, Yolcu ES, Elpek KG, Shirwan H (2010) Tumor cells engineered to codisplay on their surface 4-1BBL and LIGHT costimulatory proteins as a novel vaccine approach for cancer immunotherapy. Cancer Gene Ther 17: 730–741. 20559332 10.1038/cgt.2010.29 PMC2941532

[28] Srivastava AK, Sharma RK, Yolcu ES, Ulker V, MacLeod K, et al (2012) Prime-Boost Vaccination with SA-4-1BBL Costimulatory Molecule and Survivin Eradicates Lung Carcinoma in CD8(+) T and NK Cell Dependent Manner. PLoS One 7: e48463. 23144888 10.1371/journal.pone.0048463 PMC3493554

[29] Rabu C, Quemener A, Jacques Y, Echasserieau K, Vusio P, et al (2005) Production of recombinant human trimeric CD137L (4-1BBL). Cross-linking is essential to its T cell co-stimulation activity. J Biol Chem 280: 41472–41481. 16204238 10.1074/jbc.M506881200

[30] Elpek KG, Yolcu ES, Franke DD, Lacelle C, Schabowsky RH, et al (2007) Ex vivo expansion of CD4+ CD25+ FoxP3+ T regulatory cells based on synergy between IL-2 and 4-1BB signaling. J Immunol 179: 7295–7304. 18025172 10.4049/jimmunol.179.11.7295

[31] Vezys V, Penaloza-MacMaster P, Barber DL, Ha SJ, Konieczny B, et al (2011) 4-1BB signaling synergizes with programmed death ligand 1 blockade to augment CD8 T cell responses during chronic viral infection. J Immunol 187: 1634–1642. 21742975 10.4049/jimmunol.1100077 PMC4404506

[32] Huang CY, Chen JJ, Shen KY, Chang LS, Yeh YC, et al (2012) Recombinant lipidated HPV E7 induces a Th-1-biased immune response and protective immunity against cervical cancer in a mouse model. PLoS One 7: e40970. 22815882 10.1371/journal.pone.0040970 PMC3399806

[33] Keene JA, Forman J (1982) Helper activity is required for the in vivo generation of cytotoxic T lymphocytes. J Exp Med 155: 768–782. 6801178 10.1084/jem.155.3.768 PMC2186611

[34] Guerder S, Matzinger P (1992) A fail-safe mechanism for maintaining self-tolerance. J Exp Med 176: 553–564. 1386876 10.1084/jem.176.2.553 PMC2119333

[35] Yang Y, Xiang Z, Ertl HC, Wilson JM (1995) Upregulation of class I major histocompatibility complex antigens by interferon gamma is necessary for T-cell-mediated elimination of recombinant adenovirus-infected hepatocytes in vivo. Proc Natl Acad Sci U S A 92: 7257–7261. 7638177 10.1073/pnas.92.16.7257 PMC41318

[36] Riberdy JM, Christensen JP, Branum K, Doherty PC (2000) Diminished primary and secondary influenza virus-specific CD8(+) T-cell responses in CD4-depleted Ig(−/−) mice. J Virol 74: 9762–9765. 11000251 10.1128/jvi.74.20.9762-9765.2000 PMC112411

[37] Novy P, Quigley M, Huang X, Yang Y (2007) CD4 T cells are required for CD8 T cell survival during both primary and memory recall responses. J Immunol 179: 8243–8251. 18056368 10.4049/jimmunol.179.12.8243

[38] Janssen EM, Droin NM, Lemmens EE, Pinkoski MJ, Bensinger SJ, et al (2005) CD4+ T-cell help controls CD8+ T-cell memory via TRAIL-mediated activation-induced cell death. Nature 434: 88–93. 15744305 10.1038/nature03337

[39] Bos R, Sherman LA (2010) CD4+ T-cell help in the tumor milieu is required for recruitment and cytolytic function of CD8+ T lymphocytes. Cancer Res 70: 8368–8377. 20940398 10.1158/0008-5472.CAN-10-1322 PMC2970736

[40] Whitmire JK, Tan JT, Whitton JL (2005) Interferon-gamma acts directly on CD8+ T cells to increase their abundance during virus infection. J Exp Med 201: 1053–1059. 15809350 10.1084/jem.20041463 PMC2213135

[41] Mortarini R, Piris A, Maurichi A, Molla A, Bersani I, et al (2003) Lack of terminally differentiated tumor-specific CD8+ T cells at tumor site in spite of antitumor immunity to self-antigens in human metastatic melanoma. Cancer Res 63: 2535–2545. 12750277

[42] Feltkamp MC, Smits HL, Vierboom MP, Minnaar RP, de Jongh BM, et al (1993) Vaccination with cytotoxic T lymphocyte epitope-containing peptide protects against a tumor induced by human papillomavirus type 16-transformed cells. Eur J Immunol 23: 2242–2249. 7690326 10.1002/eji.1830230929

[43] Borowski AB, Boesteanu AC, Mueller YM, Carafides C, Topham DJ, et al (2007) Memory CD8+ T cells require CD28 costimulation. J Immunol 179: 6494–6503. 17982038 10.4049/jimmunol.179.10.6494

[44] Dolfi DV, Duttagupta PA, Boesteanu AC, Mueller YM, Oliai CH, et al (2011) Dendritic cells and CD28 costimulation are required to sustain virus-specific CD8+ T cell responses during the effector phase in vivo. J Immunol 186: 4599–4608. 21389258 10.4049/jimmunol.1001972

